# A Single Intraventricular Injection of VEGF Leads to Long-Term Neurotrophic Effects in Axotomized Motoneurons

**DOI:** 10.1523/ENEURO.0467-19.2020

**Published:** 2020-05-29

**Authors:** Paula M. Calvo, Rosa R. de la Cruz, Angel M. Pastor

**Affiliations:** Departamento de Fisiología, Facultad de Biología, Universidad de Sevilla, Sevilla 41012, Spain

**Keywords:** angiogenesis, injured motoneurons, neurotrophic factors, oculomotor system, synaptic stripping

## Abstract

Vascular endothelial growth factor (VEGF) has been recently demonstrated to induce neuroprotective and synaptotrophic effects on lesioned neurons. Hitherto, the administration of VEGF in different animal models of lesion or disease has been conducted following a chronic protocol of administration. We questioned whether a single dose of VEGF, administered intraventricularly, could induce long-term neurotrophic effects on injured motoneurons. For this purpose, we performed in cats the axotomy of abducens motoneurons and the injection of VEGF into the fourth ventricle in the same surgical session and investigated the discharge characteristics of axotomized and treated motoneurons by single-unit extracellular recordings in the chronic alert preparation. We found that injured motoneurons treated with a single VEGF application discharged with normal characteristics, showing neuronal eye position (EP) and velocity sensitivities similar to control, thereby preventing the axotomy-induced alterations. These effects were present for a prolonged period of time (50 d) after VEGF administration. By confocal immunofluorescence we also showed that the synaptic stripping that ensues lesion was not present, rather motoneurons showed a normal synaptic coverage. Moreover, we demonstrated that VEGF did not lead to any angiogenic response pointing to a direct action of the factor on neurons. In summary, a single dose of VEFG administered just after motoneuron axotomy is able to prevent for a long time the axotomy-induced firing and synaptic alterations without any associated vascular sprouting. We consider that these data are of great relevance due to the potentiality of VEGF as a therapeutic agent in neuronal lesions and diseases.

## Significance Statement

Vascular endothelial growth factor (VEGF) is a novel neurotrophic factor whose chronic administration protects motoneurons from cell death in animal models of lesion or disease. Because of the promising therapeutic perspectives of this factor, it would be interesting to know whether a single dose could produce long-lasting effects on injured motoneurons. We pursued this issue by using a behaving chronic preparation showing that single intraventricular administration of VEGF prevented the firing and synaptic alterations induced by axotomy in abducens motoneurons without provoking an angiogenic response. These data demonstrate that VEGF administration does not require a continuous supply to obtain physiological improvements, data of great value due to its potential therapeutic use.

## Introduction

Vascular endothelial growth factor (VEGF) was initially characterized by its effects on the vascular system, promoting vasculogenesis and angiogenesis (Senger et al., 1983; Ferrara and Henzel, 1989; Yancopoulos et al., 2000). Interestingly, it has also been proven to be neuroprotective. Numerous works have demonstrated its trophic effects after different types of neuronal lesions (Pan et al., 2013; Beecher et al., 2018; Calvo et al., 2018; Zeng et al., 2018; Chi et al., 2019), excitotoxic injury (Matsuzaki et al., 2001; Tovar-y-Romo et al., 2007; Tovar-y-Romo and Tapia, 2010), ischemia (Sun et al., 2003; Guo et al., 2016; Geiseler and Morland, 2018; Wang et al., 2018), epilepsy (Nicoletti et al., 2008, 2010), and neurologic diseases such as motoneuronal degeneration in amyotrophic lateral sclerosis (ALS) and spinal-bulbar muscular atrophy (Kennedýs disease), peripheral neuropathies, Alzheimer´s disease, Parkinson´s disease, demyelinating diseases, and also in traumatic spinal injury (enhancing nerve repair) and in neovascular ocular diseases (Storkebaum et al., 2005; Zachary, 2005; Wang et al., 2007, 2016; Lange et al., 2016; Guo et al., 2019). Although VEGF can act on different neuronal types (Storkebaum et al., 2004; Cabezas et al., 2019), a clear link has been established between VEGF and motoneurons (Lladó et al., 2013), mainly due to the appearance of symptoms resembling ALS when the levels of VEGF are low, as is the case for the VEGF^δ/δ^ mutant mice. These mice lack the hypoxia response element in the promotor region of the VEGF gene, so that as the animal matures the levels of VEGF decrease, leading to adult-onset motoneuron degeneration reminiscent of ALS (Oosthuyse et al., 2001). VEGF delivery to SOD^G93A^ transgenic mice (a classical model of ALS) by different gene therapies yields to a significant delay in the onset of motor deficits, slows motoneuron degeneration and prolongs survival (Azzouz et al., 2004; Wang et al., 2007, 2016). In human postmortem studies of ALS patients, the levels of VEGF and its receptors in the anterior horn cells of the spinal cord have been shown to be decreased (Brockington et al., 2006). Altogether, these works point to VEGF as a potent neurotrophic factor for motoneurons.

Previous studies supplying VEGF have delivered the factor chronically through different methods, such as osmotic pumps, viral vectors, genetic crossings with mice overexpressing VEGF, or repeated injections. Our recent work showed the effects of maintained supply of VEGF retrogradely via the proximal stump of the recessed abducens nerve (Calvo et al., 2018). Chronic VEGF retrograde supply produces the recovery of motoneuronal discharge activity and synaptic inputs altered by axotomy. In the present work, we aimed at determining the time span of the effects of VEGF after a single intraventricular application as a potential treatment route for neurologic diseases. We chose the abducens nucleus as a good model for studies of neuronal plasticity since its motoneurons can be recorded readily in the chronic alert animal preparation, and their normal discharge activity and synaptic inputs are well characterized (Delgado-Garcia et al., 1986; Escudero and Delgado-García, 1988; Escudero et al., 1992; Büttner-Ennever, 2006; Davis-López de Carrizosa et al., 2011). We sectioned the VIth nerve at the orbital level in the same surgical session as the (single) administration of VEGF into the fourth ventricle, and thereafter, we performed a neurophysiological and synaptic study of the axotomized motoneurons. We have also evaluated whether the findings obtained after VEGF intraventricular administration can be explained by its well-known angiogenic activity, or rather indicate a direct neurotrophic action on neurons. Interestingly, we have found that a single administration of VEGF intraventricularly prevents the alterations induced by axotomy on the structure/function of abducens motoneurons for a prolonged period of time, and that these effects reflect a direct neurotrophic, non-angiogenic, activity of VEGF on motoneurons.

## Materials and Methods

### Animals and surgical procedures

Experiments were performed on adult female cats weighing 2.0–2.5 kg obtained from authorized suppliers (Universidad de Córdoba, Spain). All procedures were performed in accordance with the guidelines of the European Union (2010/63/EU) and the Spanish legislation (R.D. 53/2013, BOE 34/11 370–421) for the use and care of laboratory animals and approved by the ethics committee.

A total of 11 animals was used in the present study. Three of these animals were prepared for extracellular single-unit recordings of abducens motoneurons. Motoneurons were recorded in the control situation as well as after axotomy plus VEGF treatment. The electrophysiological axotomy data were taken from our previous work by Calvo et al. (2018). In addition, in the present work we prepared eight additional animals for the morphologic study, which were not used for recordings to preserve an intact morphology: three control animals for the vascularization study (see below), two axotomized animals which were prepared exclusively for the morphologic study (one of them exclusively for the vascular study), and three animals which were axotomized and received VEGF intraventricularly. Data from two additional axotomized animals were taken from our previous study by Calvo et al. (2018).

**Figure 1.**
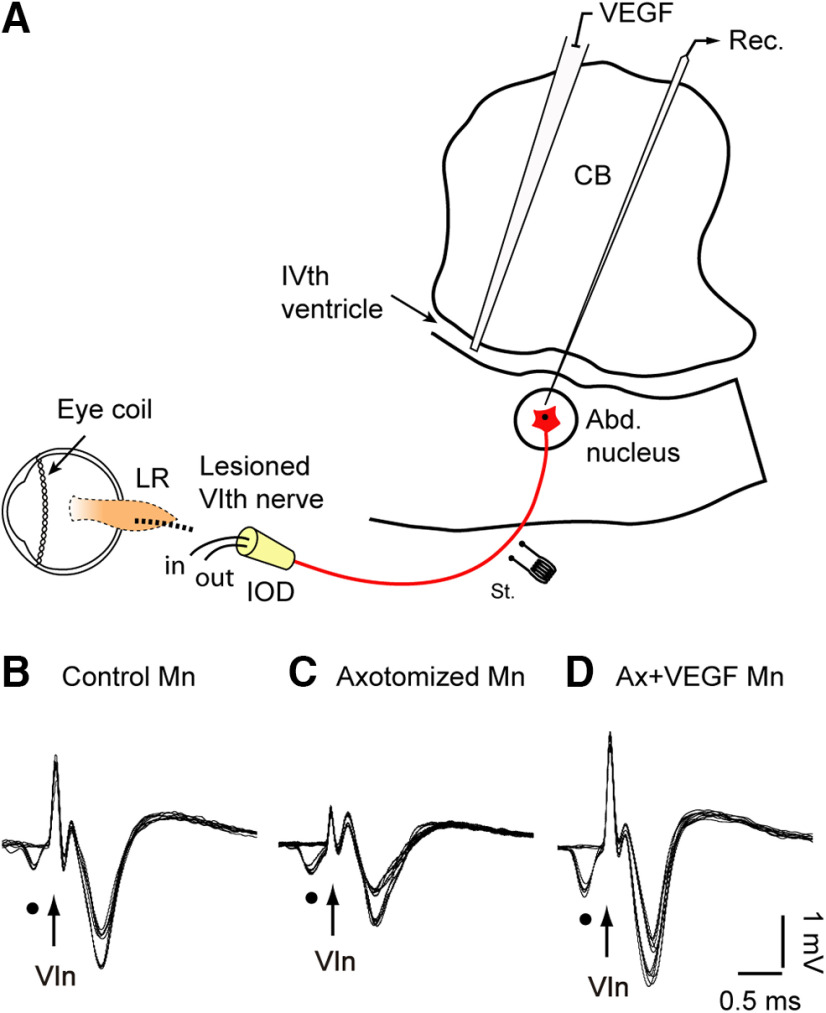
Experimental design and motoneuron identification. ***A***, Diagram illustrating the recording (Rec.) of abducens (Abd.) motoneurons identified by their antidromic activation from the electrode (St.) placed at the VIth nerve. Abd. motoneurons of the left side were axotomized at the orbital level, the lateral rectus (LR) muscle removed and the nerve proximal stump inserted into the IOD to prevent reinnervation. In the same surgical session, VEGF was delivered by pressure injection into the VIth ventricle. Recordings and VEGF infusion were conducted through the cerebellum (CB). ***B–D***, Collision tests were used for the identification of Abd. motoneurons (Mn). Three examples are shown: in ***B***, a control Mn, in ***C***, an axotomized Mn (10 d after axotomy), and in ***D***, a Mn recorded 10 d after lesion and VEGF administration. The orthodromic spontaneous spikes are indicated by dots and the arrows point to the stimulus artifact.

A detailed description of the preparation for chronic recordings has been published previously (Davis-López de Carrizosa et al., 2009, 2010). Briefly, animals received an injection of atropine sulfate (0.5 mg/kg, i.m.) to reduce vagal reflexes, then they were anesthetized with ketamine hydrochloride (20 mg/kg, i.m.) mixed with xylazine (0.5 mg/kg, i.m.) and placed in a stereotaxic frame. Two bipolar stimulating electrodes were implanted intracranially at the exit of both abducens (VIth) nerves from the brainstem (Fig. 1*A*, only left electrode illustrated) to allow the antidromic identification of abducens motoneurons. Recordings were performed in the left abducens nucleus. Coils, made up of two turns of Teflon-isolated stainless-steel wire, were implanted in the sclera of both eyes to record eye movements, and a pedestal was constructed to facilitate head-fixed extracellular recordings. A square window (5 × 5 mm) was drilled in the occipital bone to allow transcerebellar access to the brainstem for recordings (Fig. 1*A*). Postoperative care was provided daily, as needed.

### Chronic extracellular recordings

Control recording sessions started ∼10 d after surgery. The animal was gently restrained inside a fabric bag, wrapped with elastic bandages, and placed in a Perspex box (with its head immobilized), which was located inside the magnetic field for eye movement recordings (Fuchs and Robinson, 1966). Single-unit extracellular recordings were conducted with glass micropipettes filled with 2 m NaCl, attached to a three-axis micromanipulator and advanced through the intact cerebellum to reach the brainstem (Fig. 1*A*). The (left) abducens nucleus was located by recording the antidromic field potential induced by electrical stimulation of the ipsilateral VIth nerve (Delgado-Garcia et al., 1986). All recorded abducens motoneurons of the present work were identified by their antidromic activation from the VIth nerve followed by the collision test between the orthodromic and the antidromic spikes (Fig. 1*B–D*; examples for a control, an axotomized, and an axotomized + VEGF-treated motoneuron, respectively). After motoneuron identification, single-unit extracellular spikes were recorded simultaneously with eye movements under alert conditions. In some cells, the discharge activity during vestibularly-induced eye movements in the horizontal plane was also conducted. For this, the recording system rotated around the vertical axis by a servo-controlled motor attached to the turntable.

### Data storage and analysis

The horizontal eye position (EP) of both eyes and simultaneous neuronal activity were digitally stored for off-line analysis (Power 1401, Cambridge Electronic Design). For data selection, computer programs written in MATLAB 7.5 displayed instantaneous firing frequency and the position of both eyes.

As previously described (Robinson 1970; Davis-López de Carrizosa et al., 2011), the firing rate (FR) of abducens motoneurons correlates with both EP and eye velocity (EV) according to the equation FR = F_0_ + k·EP + r·EV, where k and r are the position and velocity neuronal sensitivities, respectively, and F_0_ is the FR at straight ahead gaze, i.e., when EP = 0 degrees.

FR during ocular fixations was fitted to the equation FR = F_0_ + k_s_ EP, since during eye fixations, EV is zero. The slope of the linear regression line obtained between FR and EP corresponded, therefore, to k_s_ (neuronal EP sensitivity during spontaneous eye movements, in spikes/second per degree). Relationships between neuronal FR and EV during spontaneous rapid eye movements, or saccades, were obtained by linear regression analysis, after the subtraction of the position component calculated from the previously known sensitivity to EP. Thus, the equation used was FR – k_s_·EP – F_0_ = r_s_·EV, where r_s_ represents the neuronal EV sensitivity during saccades (in spikes/second per degree/second) and represents the slope of the rate-velocity regression line.

During vestibular stimulation, the equation used was FR = F_0_ + k_v_ EP + r_v_ EV. In this case, we used multiple linear regression analysis to calculate the neuronal sensitivities k_v_ (in spikes/second per degree) and r_v_ (in spikes/second per degree/second) during vestibular eye movements. Only the slow phases of the nystagmus were selected between cursors for the analysis.

Because of the motor impairment of the eye ipsilateral to the axotomy, we used the eye movements of the contralateral (right) eye for computations. Previously, we demonstrated in control motoneurons that neuronal EP and velocity sensitivities during spontaneous and vestibularly-induced eye movements were similar when the analysis was performed with either the left or the right eye. This similarity in motoneuronal firing properties with either eye can be explained by the high degree of conjugacy in horizontal eye movements in the cat (de la Cruz et al., 2000; Davis-López de Carrizosa et al., 2009, 2010; Calvo et al., 2018).

### Axotomy and intraventricular administration of VEGF

After the postoperative recovery, we performed several control recording sessions for approximately two weeks before lesion and VEGF administration. The animal was anesthetized (ketamine hydrochloride, 20 mg/kg, i.m., mixed with xylazine, 0.5 mg/kg, i.m.) and placed in a stereotaxic frame to perform the axotomy of the VIth nerve, as previously described in detail (Davis-López de Carrizosa et al., 2008, 2009; Calvo et al., 2018). Briefly, the left VIth nerve was sectioned at its entrance into the lateral rectus muscle, which was removed, and an intraorbital device (IOD) implanted in the nerve stump. The proximal stump of the nerve was tightly introduced into the IOD, consisting in a custom-made conical chamber that had two pieces of tubing inserted for fluid replacement of the chamber with 0.01 m phosphate buffer, pH 7.4, with 0.9% saline (PBS) every 48 h. The IOD implantation also prevented the reinnervation of the severed nerve to any adjacent tissue, therefore allowing chronic axotomy.

VEGF was delivered in the fourth ventricle immediately after axotomy. We located the ventricle by lowering the recording micropipette through the cerebellum until an electrical activity silence in the recording was noticed just at the exit of the cerebellum (i.e., the IVth ventricle). Moreover, the entrance into the brainstem was also discernible by the mechanical pressure of the micropipette touching the brainstem. It is important to indicate that the abducens nucleus is located dorsally in the pons, at a distance of ∼1 mm below the floor of the fourth ventricle. For VEGF injection, we used a micropipette whose tip was beveled to a diameter of ∼20 μm. The micropipette was filled with the VEGF solution (recombinant rat VEGF_164_; R&D Systems) containing PBS and 0.1% bovine serum albumin. We delivered at this position a single application of 1 μg of VEGF in a total volume of 10 μl by means of a pressure injector. Recording sessions of about 2 h each on alternate days restarted 2–4 d after axotomy plus VEGF administration and lasted for a maximum of 50 d.

### Immunocytochemical procedures

We conducted immunofluorescence at the confocal microscopy level to evaluate the synaptic coverage in axotomized motoneurons treated with VEGF, as well as the possible angiogenic response to VEGF exogenous supply. In the three situations (control, axotomy, and axotomy plus intraventricular administration of VEGF), animals did not derive from the electrophysiological experiments.

Animals were deeply anesthetized (sodium pentobarbital, 100 mg/kg, i.p.) 21 d after injury (with or without VEGF administration) for their transcardial perfusion with physiological saline followed by fixative (4% paraformaldehyde in 0.1 m sodium phosphate buffer, pH 7.4). Following 2 h of postfixation, the brainstem at the level of abducens nucleus was cut at 50-μm-thick coronal sections on a vibratome and processed for immunocytochemistry.

Double immunofluorescence was conducted sequentially, that is, each primary antibody followed by its corresponding secondary antibody. Sections were first washed in PBS, pH 7.4, with 0.1% Triton X-100 (PBS/TX) and then blocked with normal donkey serum (1:10 in PBS/TX).

In all sections through the abducens nucleus, motoneurons were identified by their immunoreactivity against choline acetyltransferase (ChAT), obtained from goat (1:750; Millipore). Double immunofluorescence was conducted to combine ChAT immunostaining with other primary antibodies: either mouse monoclonal antibody against synaptophysin as a general marker of synaptic boutons (1:1000; Millipore), or mouse monoclonal antibody against GFAP to label astrocytes and their processes (1:400; Sigma-Aldrich).

In order to check the possibility that VEFG administration could mediate its neuronal effects, at least in part, through its well-known angiogenic activity, we characterized by immunocytochemistry the density of the vascular network in the abducens nucleus using an antibody against the glucose transporter 1 (GLUT-1), expressed selectively by endothelial cells of the blood-brain barrier (Krum et al., 2002; mouse monoclonal antibody, 1:1000; Millipore).

Secondary antibodies were obtained from Jackson ImmunoResearch, used at a dilution 1:50 in PBS/TX, and were the following: donkey anti-goat IgG coupled to TRITC (for ChAT detection) and donkey anti-mouse IgG coupled to FITC (for synaptophysin, GFAP, or GLUT-1) Finally, sections were rinsed in PBS, mounted on glass slides, and coverslipped with Dako fluorescence mounting medium (Dako North America).

Images were captured with a confocal laser scanning microscope (Zeiss LSM 7 DUO) at the same focal plane using different filters and later merged by using the Zeiss microscope software ZEN. Gray scales were adjusted to expand the maximum dynamic range of the image. Confocal images were analyzed by using the program ImageJ (NIH). For the quantification of the intensity of synaptophysin or GFAP immunostaining, two types of measurements were conducted by means of the program ImageJ, as previously described (Davis-López de Carrizosa et al., 2009, 2010; Morado-Díaz et al., 2014; Calvo et al., 2018). First, we measured the linear density of synaptophysin-immunoreactive (IR) boutons or GFAP-IR profiles surrounding the somata of ChAT-IR abducens motoneurons by using images captured at high magnification (63×) in confocal planes containing the cell nucleus. The percentage of the total perimeter of the ChAT-IR cell body occupied by synaptophysin-IR or GFAP-IR profiles was measured as an index of the linear density of the immunolabeling. Second, the intensity of synaptophysin or GFAP immunolabeling was measured also in the neuropil of the abducens nucleus using 4 × 4 tile images captured at 20×. Mean gray value (optical density) was considered as an index of the intensity of immunolabeling in the abducens neuropil (the area surrounding abducens motoneurons) and was analyzed as the average gray value of 20 square boxes of 42-μm side per image, after background subtraction. Data obtained in the neuropil were then normalized with respect to control.

To evaluate the possibility of an angiogenic response to VEGF treatment, we measured, in the three situations (control, axotomy, axotomy + VEGF), three different vascular parameters (blood vessels identified as GLUT-1-IR) in the abducens nucleus (delimited by the ChAT labeling), in all cases by stereology using a grid pattern to measure only those vessels interacting with the grid to avoid subjective bias (Croll et al., 2004; Nicoletti et al., 2008), by means of the ImageJ program. First, we measured vascular density as the area of vessels relative to the total area of the abducens nucleus (in %). Moreover, we also compared vessel lumen size between the three situations by measuring the diameter of the blood vessels, as described in detail previously (Croll et al., 2004; Nicoletti et al., 2008) and also used by others (Oosthuyse et al., 2001; Azzouz et al., 2004). A third measurement of vasculature consisted in counting the number of blood vessels as has been used previously (Oosthuyse et al., 2001), and expressed relative to unit area (2500 μm^2^) by a stereology procedure using regions of interest (ROIs) of 2500 μm^2^. The number of animals for the vasculature analysis was the following: *n *=* *3 for control, *n *=* *4 for axotomy, and *n *=* *3 for axotomy + VEGF. All quantification of fluorescent images, including synaptic, GFAP, and vascular measurements, were conducted by experimenters blind to the different groups of animals (i.e., control, axotomy, and axotomy + VEGF).

### Statistics

Results between groups were statistically contrasted by using one-way or two-way ANOVA tests, in all cases at an overall level of significance of *p *<* *0.05. ANOVA tests were followed by *post hoc* pairwise multiple comparisons using the Holm–Sidak method. The statistical program used was SigmaPlot version 11 (Systat Software). Data were expressed as mean ± SEM.

## Results

### VEGF prevents axotomy-induced alterations on motoneuron discharge during spontaneous eye movements

**Figure 2.**
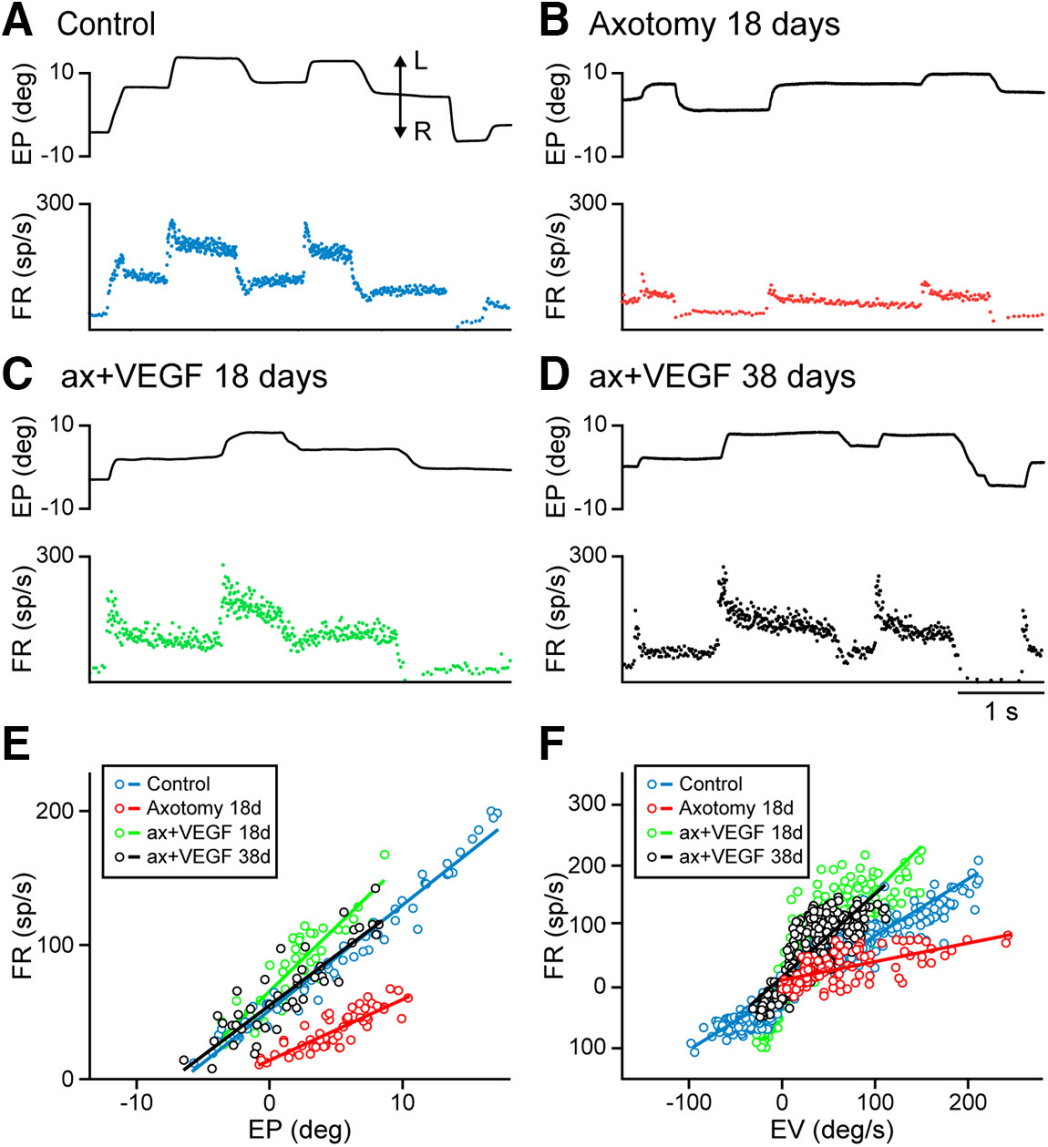
Discharge pattern of motoneurons during spontaneous eye movements. ***A***, Example of a control motoneuron showing its FR (in spikes/second) recorded simultaneously with EP (in degrees). A typical tonic-phasic discharge activity can be appreciated. Leftwards (L) and rightwards (R) eye movements are indicated by the double arrow. ***B***, Axotomized motoneurons discharged at lower rates showing the partial loss of the static and dynamic components of their firing. The illustrated cell corresponds to a motoneuron recorded 18 d after axotomy. ***C***, Example of a motoneuron recorded 18 d after axotomy + VEGF administration showing a firing pattern similar to control. ***D***, Same as ***C*** but for a motoneuron recorded 38 d after axotomy and VEGF. ***E***, Plots showing firing rate versus EP during fixations for the motoneurons shown in ***A–D***. The slope of each regression line corresponds to the EP sensitivity for that motoneuron (k_s_, in spikes/s per degree). ***F***, Regression lines obtained between FR (minus the EP component) and EV (in degrees/second) during saccades for the same four motoneurons in ***A–D***. The slope of these regression lines corresponds to the EV sensitivity (r_s_, in spikes/second per degree/second).

The discharge activity of abducens motoneurons highly correlates with EP and velocity, as previously reported (Delgado-Garcia et al., 1986; Davis-López de Carrizosa et al., 2011). Thus, a tonic firing is present during eye fixations, which increases as the eye moves toward the ipsilateral side (i.e., the on direction; in our case leftwards), and decreases for fixations in the off direction (Fig. 2*A*). Similarly, when the eye performs a rapid eye movement, or saccade, in the on-direction, motoneurons discharge a high-frequency burst of action potentials, whereas they stop firing or exhibit an abrupt drop in FR for off-directed saccades (Fig. 2*A*). Therefore, abducens motoneurons display a tonic-phasic discharge pattern during spontaneous eye movements.

The discharge of axotomized abducens motoneurons changed noticeably. Injured motoneurons discharged at lower firing frequencies than in control, during both the static and the dynamic components of the discharge (Fig. 2*B*). The absence of high-frequency bursts during on-directed saccades was very conspicuous, instead a weak increase in firing was observed during this type of eye movements (Fig. 2*B*). VEGF administration prevented all these lesion-induced alterations in axotomized motoneurons. Figure 2*C*,*D* illustrates the discharge activity of two motoneurons recorded 18 d (Fig. 2*C*) or 38 d (Fig. 2*D*) after axotomy + VEGF delivery. As can be observed, their discharge pattern resembled that of control motoneurons (compare Fig. 2*C*,*D* and *A*). It is important to emphasize that: (1) all motoneurons recorded after VEGF delivery exhibited, from a qualitative perspective, a firing pattern similar to control throughout the entire period of experimental recordings (50 d), and (2) VEGF administration was acute, that is, it was supplied only once and just at the time of axotomy.

Motoneuronal EP sensitivity (k_s_, in spikes/second per degree) was obtained as the slope of the rate-position plot during eye fixations. Linear regression lines of the rate-position plot for the four motoneurons illustrated in Figure 2*A–D*are shown in Figure 2*E*. Except for the untreated axotomized motoneuron, whose k_s_ value was low (4.5 spikes/s per degree), the motoneurons that received the single dose of VEGF, either at 18 (k_s_ = 9.6 spikes/s per degree) or 38 d post-VEGF (k_s_ = 7.48 spikes/s per degree), showed rate-position slopes and, therefore, k_s_ values, similar to the control motoneuron (k_s_ = 7.85 spikes/s per degree; Fig. 2*E*).

The same happened for the motoneuronal EV sensitivity (r_s_, in spikes/second per degree/second) during saccades, the slope of the regression line obtained between FR, previous subtraction of the EP component, and EV (Fig. 2*F*). For the motoneurons illustrated in Figure 2*A–D*, we obtained that after VEGF administration, r_s_ values (1.4 and 1.36 spikes/s per degree/s, for the motoneurons recorded at 18 and 38 d post-VEGF, respectively) were similar to control (0.93 spikes/s per degree/s), whereas the r_s_ of the axotomized motoneuron differed clearly showing a lower value (r_s_ = 0.30 spikes/s per degree/s; Fig. 2*F*).

### VEGF neurotrophic effects on axotomized motoneurons are long-lasting

**Figure 3.**
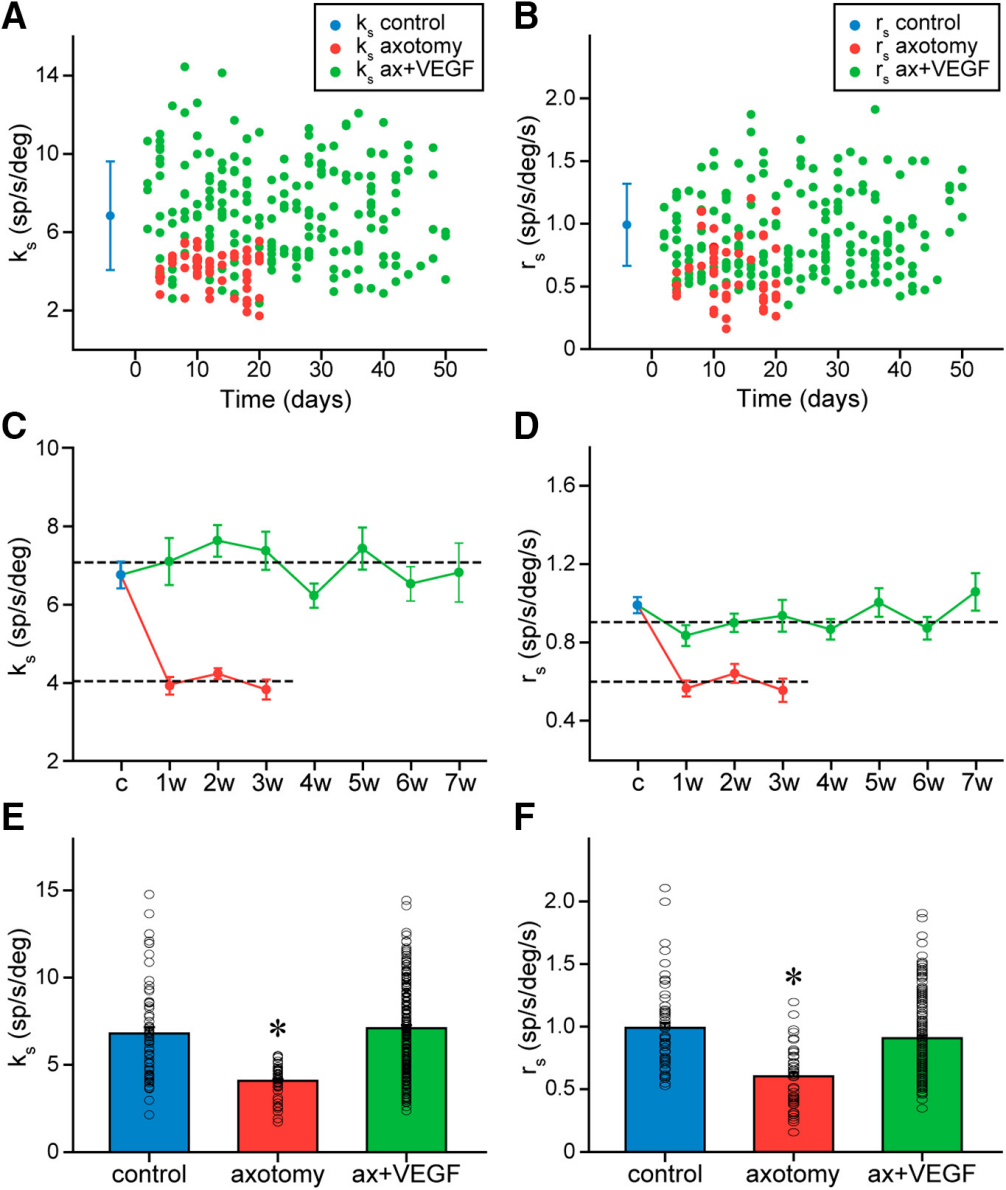
Time course of changes in EP and velocity sensitivities during spontaneous eye movements after axotomy and VEGF administration. ***A***, EP sensitivities (k_s_) of motoneurons recorded at the indicated days after axotomy (red dots; lesion corresponds to time 0) and after axotomy followed by VEGF administration (green dots). The mean control value (solid dot, in blue) and its SD (blue bars) are shown at the left of the graph. ***B***, Same as ***A***, but for EV sensitivity (r_s_). ***C***, Mean ± SEM of k_s_ values for the control population (c) and at different weeks (w) after either axotomy (in red) or axotomy plus the single VEGF delivery (in green). Horizontal dashed lines represent the a alone (lower line). There were significant differences between axotomy and VEGF groups comparing per week (two-way ANOVA, *p *<* *0.001). ***D***, Same as ***C***, but for r_s_. No significant differences were found between the mean values obtained per week after the supply of VEGF for k_s_ (***C***; one-way ANOVA, *p *=* *0.275) or r_s_ (***D***; one-way ANOVA, *p *=* *0.201). For ***C***, ***D***, the number of motoneurons recorded at the different weeks after treatment ranged between 12 and 40, and for axotomy between 8 and 28; *n *=* *64 control motoneurons. ***E***, Bar chart illustrating k_s_ data and its mean value for in the population of motoneurons recorded in the control situation, after only axotomy and after axotomy plus VEGF administration. The asterisk indicates significant difference between the axotomy and control groups (one-way ANOVA, *p *<* *0.001). On the contrary, the VEGF-treated group showed no significant difference with control (*p *=* *0.437). ***F***, Same as ***E***, but for r_s_ values. The axotomy group showed a significant difference in r_s_ with respect to control (asterisk, one-way ANOVA, *p *<* *0.001), whereas the VEGF-treated group was similar to control (*p *=* *0.064). For ***E***, ***F***, the number of motoneurons analyzed in each situation was *n *=* *64, 57, and 199 for the control, axotomy, and VEGF-treated groups, respectively.

The time course of changes in k_s_ and r_s_ in motoneurons was evaluated in the two experimental conditions: axotomy only and axotomy plus a single dose of intraventricular VEGF. As a first approximation, we elaborated a graph displaying over time the values of k_s_ (Fig. 3*A*) and r_s_ (Fig. 3*B*) of the motoneurons recorded in each condition. It can be observed that the great majority of k_s_ and r_s_ data in the VEGF-treated motoneurons appeared distributed within the mean ± SD of the control group of motoneurons (Fig. 3*A*,*B*). In contrast, axotomized motoneurons presented lower k_s_ and r_s_ values, which in many cases appeared below the mean minus 1 SD of control data. Although our data of axotomy were obtained only during 21 d after lesion (Calvo et al., 2018), it has been previously described that chronic axotomy produces long-lasting changes as long as reinnervation is not allowed (Delgado-Garcia et al., 1988). Therefore, as a first approximation, these data showed similar k_s_ (Fig. 3*A*) and r_s_ (Fig. 3*B*) values in the VEGF-treated motoneurons as compared with the control cells and, in addition, this similarity remained throughout the experiment.

To further evaluate whether the single administration of VEGF led to a long-time recovery, motoneurons were grouped in week intervals beginning from the day of surgery (i.e., the day when axotomy plus VEGF intraventricular injection was performed) until the last week of recordings (seventh week), and mean k_s_ and r_s_ values were calculated per week and compared with control (Fig. 3*C*,*D*). The overall comparison of the k_s_ values obtained per week after treatment along with the control data revealed similarity between groups (one-way ANOVA, *F*_(7,253)_ = 1.251, *p *=* *0.275; *n* ranged between 12 and 40 motoneurons per week; for control *n *=* *64), therefore indicating the absence of any change in k_s_ over time, and similarity of all groups in relation to control (Fig. 3*C*). In contrast, axotomy data grouped per week were significantly lower than the VEGF data for the same week [for k_s_: two-way ANOVA, *F*_(5,141)_ = 69.465, *p *<* *0.001 (Fig. 3*C*); for r_s_: two-way ANOVA, *F*_(5,141)_ = 28.732, *p *<* *0.001 (Fig. 3*D*)]. Of especial relevance was the fact that k_s_ did not fall toward lower values at long periods after VEGF application, and therefore motoneuronal EP sensitivity did not return to the axotomy situation. For r_s_ values, we obtained similar results. Thus, the data grouped per week plus the control group were compared with each other, and the results of this comparison revealed absence of any statistically significant difference (one-way ANOVA, *F*_(7,253)_ = 1.341, *p *=* *0.231; same *n* as for k_s_ data, see above). Therefore, as can be appreciated in Figure 3*D*, there was no tendency in r_s_ values to decrease with time after treatment.

Since no differences were found in k_s_ and r_s_ over time, we grouped all motoneurons recorded during each situation: control (*n *=* *64), axotomy alone (*n *=* *57), or axotomy plus VEGF delivery (*n *=* *199), and calculated their respective k_s_ and r_s_ mean values. The results obtained indicated statistically significant differences [k_s_: one-way ANOVA, *F*_(2,317)_ = 35.674, *p *<* *0.001 (Fig. 3*E*); r_s_: one-way ANOVA, *F*_(2,317)_ = 26.196, *p *<* *0.001 (Fig. 3*F*)] so that k_s_ and r_s_ of axotomized motoneurons were significantly (*p *<* *0.001) lower than control, whereas the values obtained after the administration of VEGF were similar to control (*p *=* *0.437 for k_s_ and *p *=* *0.072 for r_s_). Altogether, these results indicated that a single dose of VEGF prevented for a long time the changes induced by axotomy in the EP and velocity sensitivities of motoneurons during spontaneous eye movements.

### Vestibular signals remain normal in axotomized motoneurons after a single dose of VEGF

**Figure 4.**
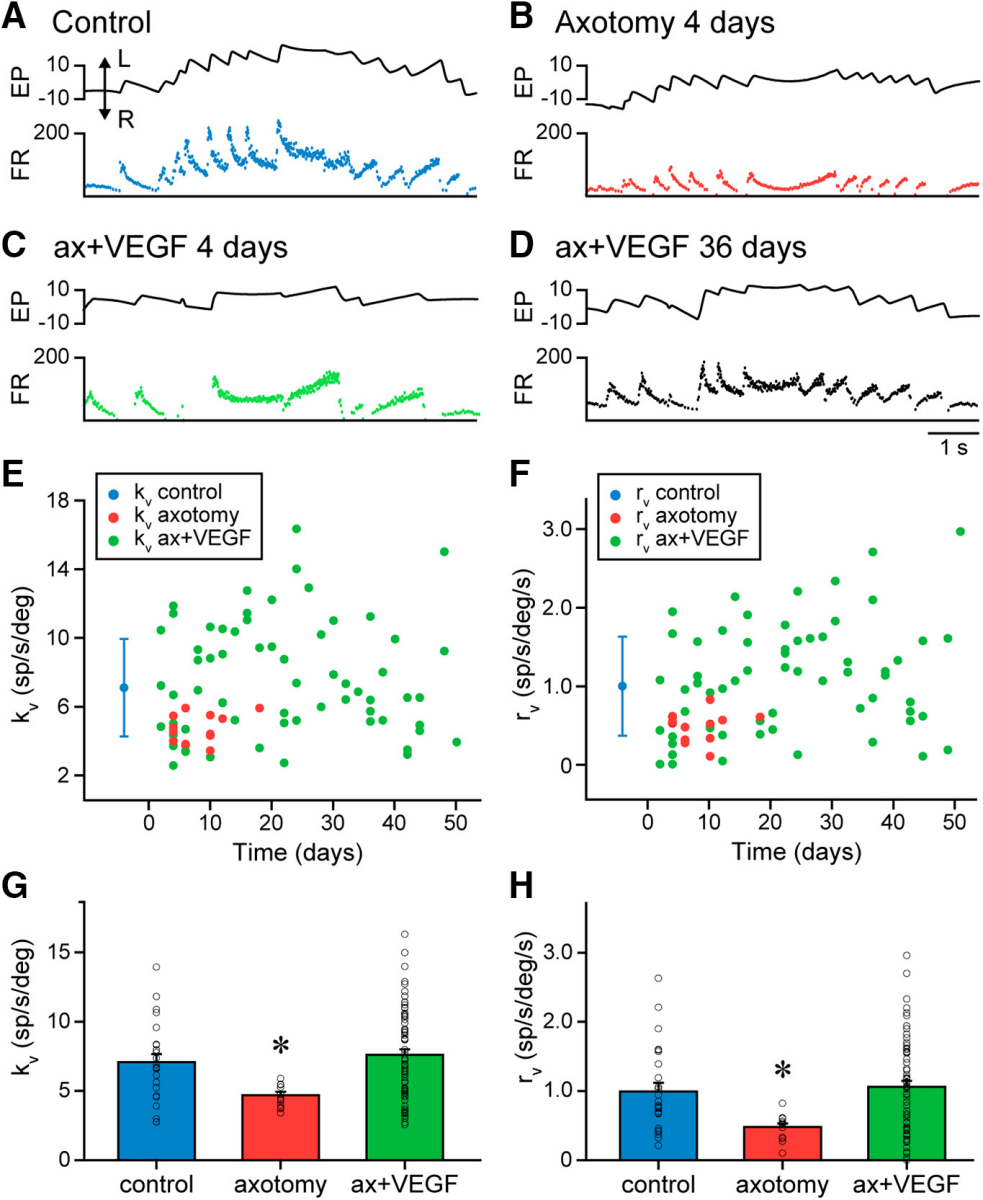
Behavior of abducens motoneurons during vestibularly-induced eye movements. ***A–D***, Examples of motoneurons recorded during vestibular eye movements in control (***A***), axotomy (***B***; 4 d after lesion), and after VEGF administration (***C***; 4 d and ***D***; 36 d). Traces correspond to EP (in degrees) and FR (in spikes/second). Eye movements to the left (L) are upwards in the EP trace (double arrow). ***E***, ***F***, Time course of changes in EP (k_v_; ***E***) and EV (r_v_; ***F***) during vestibular eye movements. Control data (mean ± SD) are indicated by a blue dot and bars. k_v_ and r_v_ values for the axotomized population are indicated by red dots in ***E***, ***F***, respectively, as a function of time following the day of lesion (day 0). For VEGF-treated motoneurons, k_v_ data are illustrated in ***E*** (green dots) and those of r_v_ in ***F*** (green dots) throughout time after the day of surgery (lesion plus VEGF administration, day 0). ***G***, ***H***, All the population of control (*n *=* *25), axotomized (*n *=* *13) or VEGF-treated motoneurons (*n *=* *64) recorded during vestibular eye movements were grouped in three pools, and their EP (k_v_; ***G***) and EV (r_v_; ***H***) sensitivities were compared. For k_v_, axotomized motoneurons showed a significantly lower sensitivity as compared with control (***G***; asterisk; one-way ANOVA, *p *=* *0.021), whereas similar k_v_ values were found between control and VEGF-treated motoneurons (***G***; *p *=* *0.470). The same happened for r_v_ (in ***H***): axotomized motoneurons presented a significantly lower value than control (asterisk; one-way ANOVA, *p *=* *0.021), but motoneurons of the VEGF group showed r_v_ values similar to control (*p *=* *0.657).

During vestibularly-induced eye movements produced by horizontal rotation of the table, abducens motoneurons discharged in correlation with EP and EV as previously described (Delgado-Garcia et al., 1986; Fuchs et al., 1988; Davis-López de Carrizosa et al., 2009, 2010). Control motoneurons increased their discharge for ipsilateral eye movements during both the slow and the fast phases of the vestibulo-ocular reflex, and reduced their FR for eye movements in the opposite direction (Fig. 4*A*). In contrast, axotomy led to a significant reduction in motoneuronal discharge during vestibular eye movements (Fig. 4*B*). When VEGF was applied in the fourth ventricle immediately after axotomy, motoneurons recorded thereafter showed a normal discharge pattern during vestibular eye movements that resembled the pattern observed in control. Axotomized VEGF-treated motoneurons behaved normally throughout the whole period of recordings after VEGF administration (50 d), as illustrated for the two examples of motoneurons recorded 4 and 36 d after VEGF administration (Fig. 4*C*,*D*, respectively).

We analyzed EP and EV sensitivities (k_v_ and r_v_, respectively) during the slow phases of the nystagmus in control, axotomized and VEGF-treated motoneurons. As shown in Figure 4*E*,*F*, k_v_ and r_v_ values of axotomized cells were low in comparison with control (mean ± SD), whereas those of injured motoneurons that received VEGF appeared similar to control. In addition, it can be observed that these parameters were maintained within a normal range in the VEGF-treated motoneurons throughout the whole period of recordings. Pooling the motoneurons per week after VEGF delivery, we found no significant difference between the seven VEGF groups (from the first to the seventh week) and control (k_v_: one-way ANOVA, *F*_(7,81)_ = 1.442, *p *=* *0.2; r_v_: one-way ANOVA, *F*_(7,81)_ = 1.535, *p *=* *0.167; *n* ranged between 5 and 13 treated motoneurons per week, and for the control group *n *=* *25; not illustrated). Therefore, we clustered the motoneurons in three groups: control, axotomy alone, and axotomy plus VEGF delivery. Comparison between groups revealed significant differences between groups [for k_v_: one-way ANOVA, *F*_(2,99)_ = 5.121, *p *=* *0.008 (Fig. 4*G*); for r_v_: one-way ANOVA, *F*_(2,99)_ = 4.490, *p *=* *0.014; *n *=* *25, 13, and 64 motoneurons for control, axotomy, and VEGF groups, respectively (Fig. 4*H*)]. In particular, axotomized motoneurons presented significantly lower k_v_ (*p *=* *0.021; Fig. 4*G*) and r_v_ (*p *=* *0.021; Fig. 4*H*) values than control, whereas injured motoneurons treated with VEGF had k_v_ (*p *=* *0.470; Fig. 4*G*) and r_v_ (*p *=* *0.657; Fig. 4*H*) values similar to control cells. Therefore, as happened for spontaneous eye movements, a single dose of VEGF applied to axotomized motoneurons was able to prevent the alterations in motoneuronal sensitivities to EP and EV during vestibularly-induced eye movements for a prolonged period of time.

### Synaptic density is preserved in the long-term after a single pulse of VEGF to axotomized motoneurons

**Figure 5.**
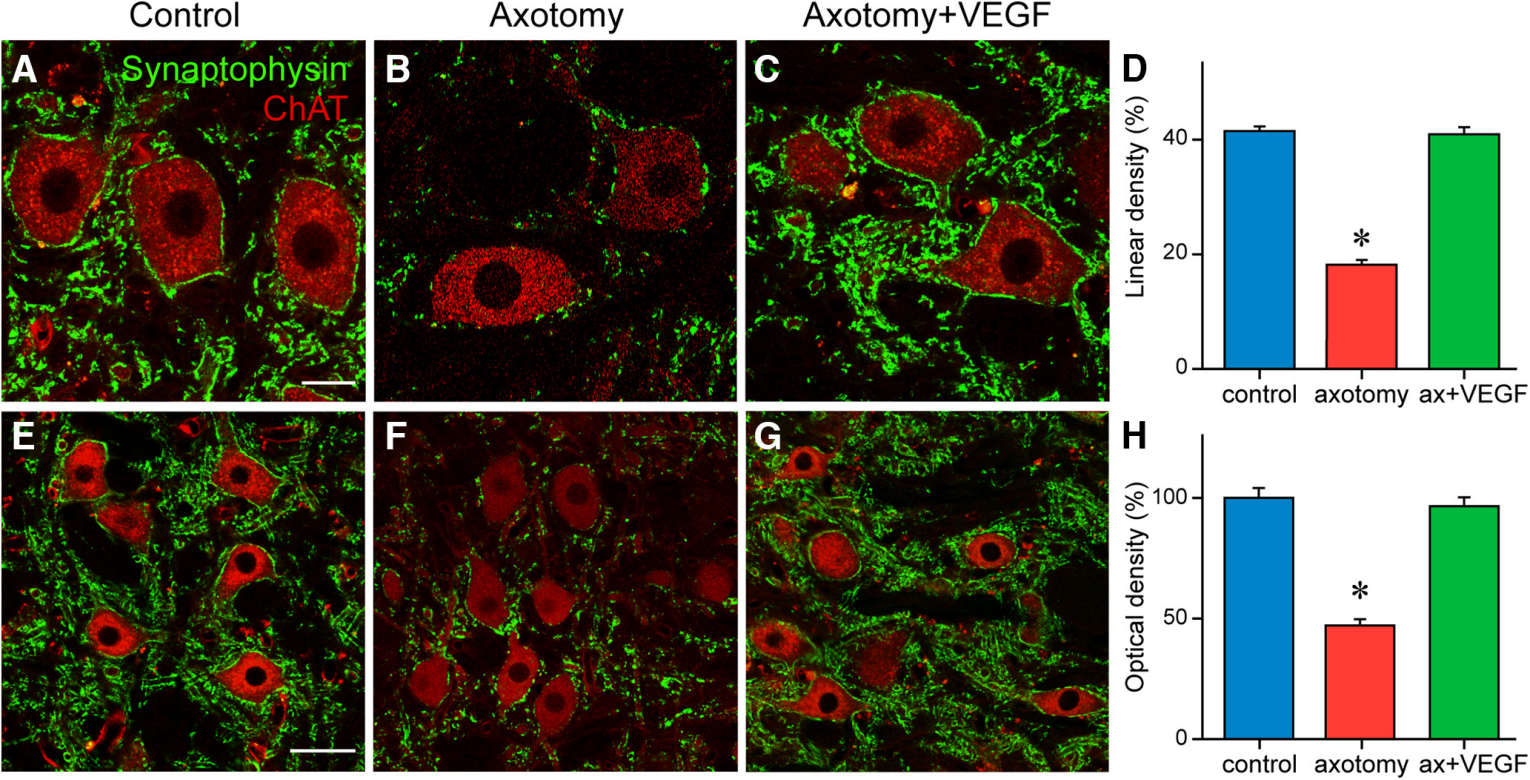
VEGF prevents the synaptic loss induced by axotomy. Confocal images obtained after double immunofluorescence against ChAT, to label the motoneurons, and synaptophysin, to label synaptic boutons, around cell bodies (***A–C***) and in the neuropil (***E–G***). Images are shown for control (***A***, ***E***), axotomy (***B***, ***F***), and after axotomy + VEGF supply (***C***, ***G***). ***D***, Bar chart illustrating the percentage of the somatic perimeter of motoneurons covered by synaptophysin-positive boutons (i.e., linear density) in the three situations. The statistical analysis revealed a significant (*p *<* *0.001) decrease in synaptic coverage after axotomy as compared with control (asterisk), whereas data obtained after VEGF delivery were similar (*p *=* *0.703) to control (one-way ANOVA). ***H***, Same as ***D*** but for measurements of synaptophysin optical density in the neuropil of the abducens nucleus. Axotomy induced also a significant (*p *<* *0.001) decrease (asterisk) in synaptic boutons at the neuropil level, but VEGF administration prevented this reduction producing values similar (*p *=* *0.503) to control (one-way ANOVA). For ***D***, ***H***, mean ± SEM are illustrated. Scale bars: 20 μm in ***A*** (for ***A–C***) and 50 μm in ***E*** (for ***E*–*G***).

Synaptophysin immunoreactivity was used as a general marker of synaptic boutons. We quantified the percentage of the somatic perimeter of abducens motoneurons that appeared contacted by synaptophysin-positive boutons, an index of linear density (in %) 21 d after lesion (with or without VEGF). A simple inspection of synaptophysin-IR boutons in the three situations revealed that control motoneurons presented a high density of perisomatic boutons (Fig. 5*A*) that contrasted with the low synaptophysin-IR boutons contacting axotomized motoneurons (Fig. 5*B*). In contrast, VEGF-treated motoneurons showed a normal density of synaptophysin-positive boutons contacting their somatic perimeter that resembled that found in control (Fig. 5*C*). Quantification of the linear density of synaptophysin-immunostained terminals (Fig. 5*D*) revealed significant differences between groups (one-way ANOVA, *F*_(2,191)_ = 144.762, *p *<* *0.001). Thus, control motoneurons (*n *=* *69) presented a somatic synaptic coverage of 41.44 ± 0.84%, whereas in axotomized cells (*n *=* *54), synaptic coverage fell to 18.12 ± 0.97%, which resulted in a significant difference (*p *<* *0.001). In contrast, injured cells that received a single dose of VEGF showed 21 d later (*n *=* *71) a normal synaptic coverage (40.90 ± 1.24%) around their cell bodies that was similar to control (*p *=* *0.703; Fig. 5*D*).

A quantification of the intensity of synaptophysin immunoreactivity (i.e., optical density, in percentage with respect to control) was also performed at the level of the neuropil (the area between labeled motoneurons). Results were similar to those described above for the cell bodies. Therefore, neuropil immunostaining of synaptophysin after treatment with VEGF (Fig. 5*G*) was similar to control (*p *=* *0.503; Fig. 5*E*,*H*), in contrast to the lower intensity obtained in the injured abducens neuropil (*p *<* *0.001; Fig. 5*F*). The statistical analysis confirmed these observations (one-way ANOVA, *F*_(2,417)_ = 58.371, *p *<* *0.001; *n *=* *180, 120, and 120 for control, axotomy, and VEGF, respectively; Fig. 5*H*). The finding that abducens motoneurons and their neuropil showed a normal synaptic density 21 d after injury and VEGF application was in good agreement with our physiological data.

### The astroglial reaction induced by axotomy is prevented by a single VEGF administration

**Figure 6.**
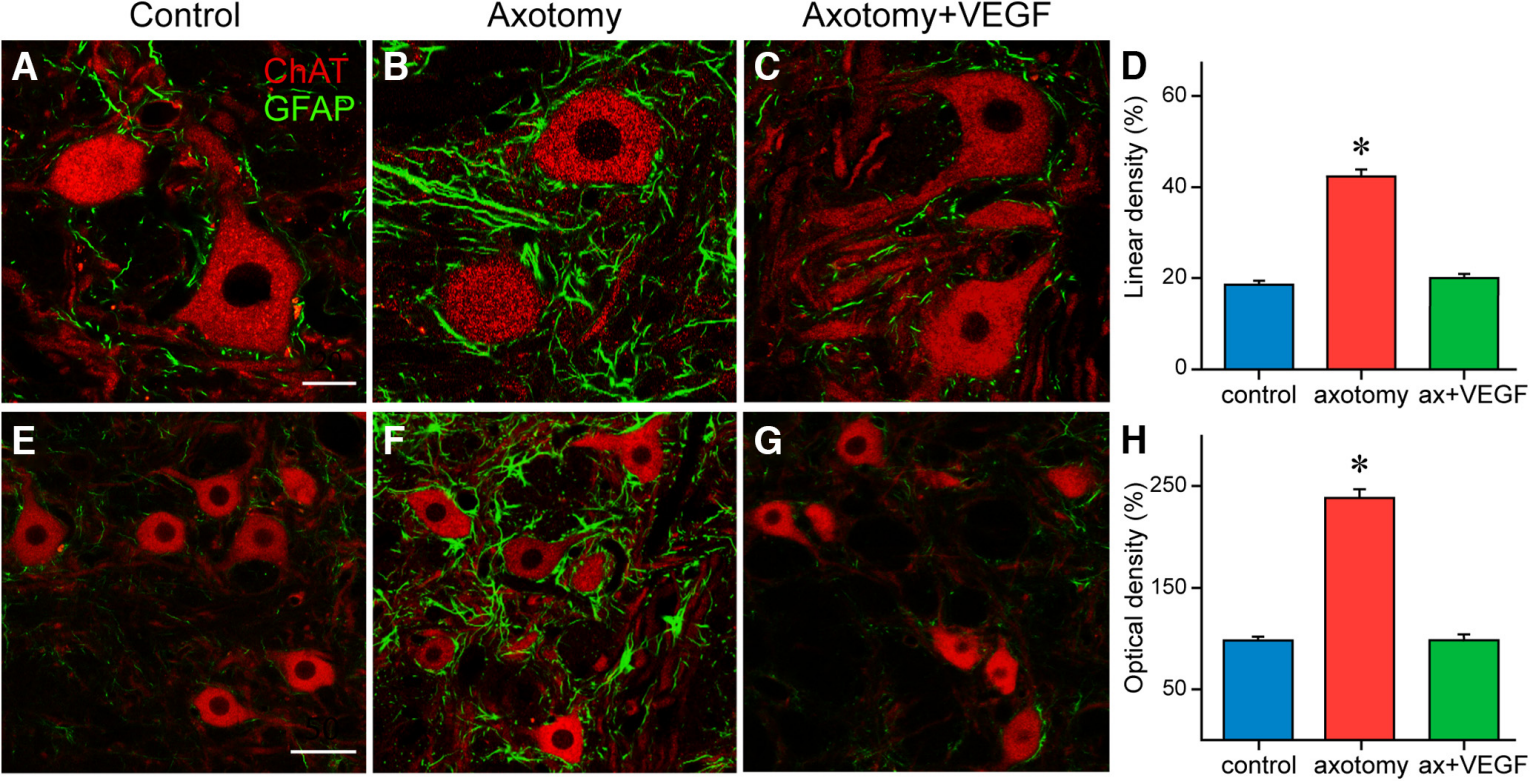
VEGF abolishes the astroglial reaction induced by axotomy. Confocal images of double immunofluorescence for GFAP, as a marker of astrocytes, and ChAT, as a marker of motoneurons, both at the level of the cell bodies (***A–C***) and in the abducens neuropil (***E–G***). The three situations are shown: control (***A***, ***E***), axotomy (***B***, ***F***), and post-VEGF (***C***, ***G***). ***D***, ***H***, Bar charts illustrating the percentage of motoneuron somata covered by GFAP-IR profiles (i.e., lineal density; ***D***), and the intensity of GFAP immunostaining (i.e., optical density; ***H***) in the abducens neuropil. After axotomy, there was a significant increase (asterisk) in both lineal (***D***; *p *<* *0.001) and optical (***H***; *p *<* *0.001) density as compared with control data. However, VEGF delivery blocked the astroglial response to lesion, since values obtained after VEGF were similar (*p *=* *0.349; ***D*** and *p *=* *0.963; ***H***) to control (one-way ANOVA). Data correspond to mean ± SEM. Scale bars: 20 μm in ***A*** (for ***A–C***) and 50 μm in ***E*** (for ***E*–*G***).

GFAP immunostaining was used to characterize whether VEGF was able to prevent the glial reaction typically found around axotomized motoneurons. This analysis was performed 21 d after lesion (with or without VEGF delivery). Measurements were done as described above for synaptophysin. Thus, we determined first the percentage of the somatic perimeter of ChAT-identified motoneurons covered by GFAP-profiles in the three situations, i.e., control (*n *=* *68), axotomy alone (*n *=* *55), and axotomy plus VEGF (*n *=* *83). Images revealed a marked increase in GFAP-IR processes surrounding motoneuronal cell bodies in the axotomy condition (Fig. 6*B*) in comparison to the low GFAP staining around control (Fig. 6*A*) and VEGF-treated cells (Fig. 6*C*). The statistical analysis confirmed these observations (one-way ANOVA, *F*_(2,203)_ = 126.267, *p *<* *0.001; Fig. 6*D*). Thus, there was a significant increase (*p *<* *0.001) in GFAP linear density around axotomized motoneuron somata (42.37 ± 1.56%) with respect to control (18.69 ± 0.89%), whereas control and VEGF data (20.09 ± 0.96%) were similar (*p *=* *0.349).

Second, measurements of GFAP immunostaining intensity (an index of optical density) at the neuropil level, also revealed an increase in GFAP-IR profiles in the abducens neuropil of axotomized animals (Fig. 6*F*) in comparison to control (Fig. 6*E*). In contrast, the appearance of GFAP immunostaining in the abducens nucleus neuropil after VEGF intraventricular delivery (Fig. 6*G*) resembled that of control (Fig. 6*E*). We compared the optical density for GFAP in the three situations by using the one-way ANOVA test (*F*_(2,517)_ = 155.835, *p *<* *0.001; *n *=* *220, 180, and 120 for control, axotomy, or axotomy plus VEGF, respectively). The statistical comparison revealed a significant (*p *<* *0.001) increase in GFAP optical density in the axotomy situation versus control. However, GFAP optical density was similar (*p *=* *0.963) between control and VEGF groups. These results indicated that a single administration of VEGF was able to inhibit the astroglial reaction typically found after axotomy.

### Intraventricular delivery of VEGF is not followed by angiogenesis in the abducens nucleus

**Figure 7.**
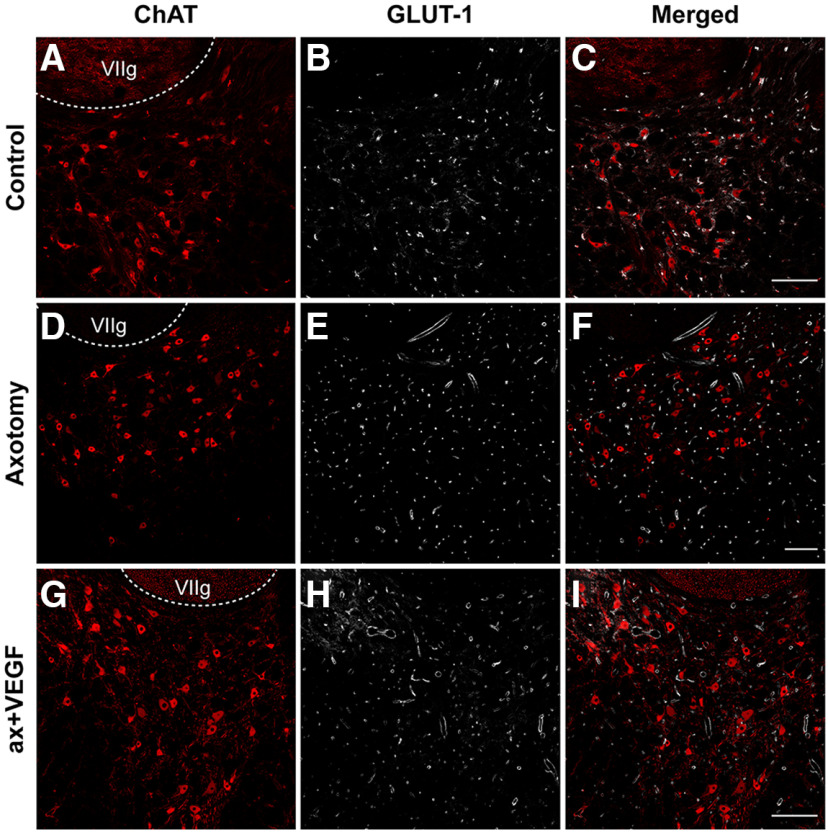
Absence of angiogenic response after VEGF delivery. Confocal images through the abducens nucleus following double immunofluorescence against GLUT-1 (a marker of endothelial cells used to label blood vessels; ***B***, ***E***, ***H***) and ChAT (a marker of motoneurons; ***A***, ***D***, ***G***) in control (***A–C***), axotomy (***D–F***), and after VEGF application (***G–I***). The merged images of GLUT-1 and ChAT immunostaining are shown for each situation in ***C***, ***F***, ***I***, respectively. Note the similarity in the density of blood vessels between control, axotomy, and VEGF intraventricular injection. The genu of the facial nerve is indicated (VIIg). Scale bars: 200 μm in ***C*** (for ***A–C***), 200 μm in ***F*** (for ***D–F***), and 200 μm in ***I*** (for ***G–I***).

Since VEGF promotes the formation of new blood vessels when administered in the brain, although in doses higher than the one used here (Manoonkitiwongsa et al., 2004), we investigated whether the injection of 1 μg of VEGF into the fourth ventricle induced an angiogenic response in the abducens nucleus. For this purpose, GLUT-1 antibody was used as a selective marker of endothelial cells. GLUT-1 was combined in double immunofluorescence with ChAT to label the motoneurons and delimit the boundaries of the abducens nucleus. GLUT-1 was a good marker of blood vessels, as can be observed in the images of Figure 7. The appearance of the abducens nucleus in control (Fig. 7*A–C*), 21 d after axotomy alone (Fig. 7*D–F*), or 21 d after axotomy plus VEGF supply (Fig. 7*G–I*) was similar with respect to the density of blood vessels.

A quantification was performed to compare the vasculature in the abducens nucleus between the three situations. We conducted three different vascular measurements. First, vascular density was measured within the limits of the abducens nucleus (delimited by ChAT immunolabeling) using stereological methods (see Materials and Methods). As can be observed in Table 1, there were no significant differences in vascular density between control, axotomy, and axotomy plus VEGF (one-way ANOVA, *F*_(2,26)_ = 2.466, *p *=* *0.105). Second, the diameter of blood vessels was also measured and again we found absence of significant differences between the three situations (one-way ANOVA, *F*_(2,26)_ = 2.210, *p *=* *0.130; Table 1). Finally, as a third procedure to measure vasculature we counted the number of blood vessels per 2500 μm^2^ in control, axotomy, and axotomy + VEGF. No significant differences were obtained in this parameter between the three situations (one-way ANOVA, *F*_(2,26)_ = 2.862, *p *=* *0.075; Table 1). Therefore, the present findings indicated that the administration of VEGF did not induce any angiogenic response in the abducens nucleus. Moreover, since VEGF was injected into the VIth ventricle, we also measured the vascular network in another brainstem structure, to verify that the result found in the abducens nucleus was general and that indeed VEGF did not induce the growing of blood vessels in the brain. Thus, we also measured the same three vascular parameters as for the abducens nucleus in the medial vestibular nucleus, and in the same three situations (control, axotomy, axotomy + VEGF). For the medial vestibular nucleus, vascular density (one-way ANOVA, *F*_(2,13)_ = 0.233, *p *=* *0.795; *n *=* *6, 5, and 5, for control, axotomy, and axotomy + VEGF, respectively), blood vessel diameter (one-way ANOVA, *F*_(2,13)_ = 0.648, *p *=* *0.539; *n *=* *6, 5, and 5, for control, axotomy, and axotomy + VEGF, respectively) and number of vessels/2500 μm^2^ (one-way ANOVA, *F*_(2,13)_ = 2.159, *p *=* *0.155; *n *=* *6, 5, and 5, for control, axotomy, and axotomy + VEGF, respectively) were similar between control, axotomy, and axotomy plus VEGF. Therefore, the data found in the medial vestibular nucleus reinforced those found at the level of the abducens nucleus, indicating that there was no angiogenic response around abducens motoneurons following the administration of VEGF into the IVth ventricle.

**Table 1:** Quantification of the vasculature in the abducens nucleus (mean ± SEM)

	*n*	Control	Axotomy	Axotomy + VEGF
Vascular density (%)	10	1.95 ± 0.30	2.80 ± 0.45	1.87 ± 0.17
Vessel diameter (μm)	9	9.22 ± 0.48	9.53 ± 0.47	8.34 ± 0.27
No. vessels/2500 μm^2^	10	4.69 ± 0.26	4.02 ± 0.25	4.73 ±0.17

For the analysis of the vasculature in the abducens nucleus in the three situations (control, axotomy, and axotomy + VEGF), we calculated by stereology: (1) vascular density relative to the area of abducens nucleus (in %); (2) the size of blood vessels by measuring their diameter (in μm); and (3) the number of vessels per 2500 μm^2^. Blood vessels were identified by GLUT-1 immunoreactivity, and the area of the abducens nucleus was delimited by ChAT immunolabeling. In no case, significant differences were obtained between the three situations (ANOVA test; *p *=* *0.105 for vessel area, *p *=* *0.130 for vessel diameter, and *p *=* *0.075 for number of vessels per 2500 μm^2^).

## Discussion

The present work has shown that a single intraventricular administration of VEGF prevents the changes induced by axotomy on the physiological properties and synaptic inputs of abducens motoneurons for a prolonged period of time. Moreover, our data indicate that the beneficial effects of VEGF were not due to its angiogenic activity, but to a direct neurotrophic action of this factor on motoneurons.

### A single administration of VEGF produces long-lasting effects in axotomized abducens motoneurons

Axotomy of abducens motoneurons is characterized by a general decrease in FR that affects both the tonic and the phasic components of the discharge (Delgado-Garcia et al., 1988; Davis-López de Carrizosa et al., 2009, 2010; Calvo et al., 2018). We have delivered a single injection of VEGF into the IVth ventricle of cats in the same surgical session as the axotomy. The administration of VEGF prevented the appearance of the axotomy effects, as shown by the recordings performed from the second day onwards after VEGF injection. A striking finding of the present work was that motoneurons continued firing with normal characteristics up to 50 d (the maximum time interval studied) after VEGF administration.

This finding contrasts markedly with those obtained previously in our model using other neurotrophic factors, in particular, the neurotrophins brain-derived neurotrophic factor (BDNF), neurotrophin-3 (NT-3), or nerve growth factor (NGF; Davis-López de Carrizosa et al., 2009, 2010). In those works, the removal of the factor produced the rapid reappearance of the lesion-induced alterations in firing pattern, which indicated that the delivery of BDNF, NT-3, or NGF required continuity over time for the maintenance of their trophic effects. Moreover, these factors produced only partial recovery in the discharge of axotomized abducens motoneurons, in contrast to the complete recovery observed in the present work after VEGF treatment, which points to VEGF as a more powerful neurotrophic factor than neurotrophins for this motoneuronal type.

A possible mechanism to explain the long-term effects of a single administration of VEGF found in the present work might be related to its heparin-binding capability. VEGF has a heparin-binding domain that allows this factor to interact with the heparin-sulfate-rich extracellular matrix, and therefore to remain for a long period of time within the tissue, prolonging its biological activity (Robinson and Stringer, 2001; Tokunaga et al., 2005; Krilleke et al., 2009).

In agreement with our results, previous studies have also shown long-term effects after the single administration of a neurotrophic factor. This has been analyzed extensively in the hemiparkinsonian rat model, induced by the unilateral intrastriatal injection of 6-hydroxydopamine to lesion the nigrostriatal dopaminergic system. A single administration of glial cell line-derived neurotrophic factor (GDNF) to these rats produces behavioral recovery, and increases both the number of substantia nigra neurons and the density of striatum fibers, that last several weeks (12 weeks) after the removal of the factor (Hudson et al., 1995; Kearns and Gash, 1995; Sullivan et al., 1998; Aoi et al., 2000; Yue et al., 2014). Using also the hemiparkinsonian rat model, two novel neurotrophic factors [cerebral dopamine neurotrophic factor (CDNF) and mesencephalic astrocyte-derived neurotrophic factor (MANF)] have also been shown to induce, following their single injection into the striatum, long-lasting (12 weeks) recovery in motor performance and in the number of substantia nigra neurons and striatum fiber density (Lindholm et al., 2007; Voutilainen et al., 2009; Airavaara et al., 2012). Using genetic engineering to turn “on” or “off” BDNF expression, it has also been demonstrated that transient growth factor delivery is sufficient to sustain regenerated axons for prolonged time periods in spinal cord lesion models (Blesch and Tuszynski, 2007). Moreover, studies that have compared single administration with continuous infusion of BDNF or NGF have revealed no difference between both patterns of delivery (Knusel et al., 1996; Chai et al., 1999).

The finding that the discharge of axotomized plus VEGF-treated motoneurons was maintained normal suggests that both electrical membrane properties and synaptic inputs were not affected. Neurotrophic factors are known to regulate voltage-gated ion channels, therefore affecting neuronal excitability (Lesser et al., 1997; Blum et al., 2002; Tucker and Fadool, 2002; Mitre et al., 2017). In this regard, it is important to point out that a single pulse of NGF produces long-term neuronal excitability through the induction of sodium channels (Toledo-Aral et al., 1995). Neurotrophic factors are also involved in the formation and stabilization of synapses (Vicario-Abejón et al., 2002), and in the structural and functional changes that operate during synaptic plasticity (McAllister et al., 1999; Poo, 2001; Tyler and Pozzo-Miller, 2001; Elmariah et al., 2004; Kowiański et al., 2018). BDNF plays also an important role in long-term potentiation in the hippocampus (Minichiello, 2009), enhancing the efficacy of synaptic transmission and modulating synaptic structures, including increased number of dendritic spines, changes that are of long duration (Poo, 2001; Castrén, 2013; Park and Poo, 2013).

### Synaptic stripping and astroglial reaction in axotomized abducens motoneurons are prevented by a single dose of VEGF

We also investigated by confocal immunofluorescence the density of synaptic boutons and the astrocytic reaction surrounding motoneurons after both axotomy alone and axotomy + VEGF treatment, to elucidate whether there was a correlation between physiological data and synaptic coverage. This study was performed 21 d postsurgery.

Following axonal injury, there is a general retrograde response at the cell body level that includes the removal of synapses (the so-called synaptic stripping) from the somata and dendrites of lesioned cells (Svensson et al., 1993; Cullheim and Thams 2007; Thams et al., 2008). Astrocytes and microglia play an important role in this process since they experience hypertrophy with increased level of GFAP and proliferation, respectively (Svensson et al., 1993; Pastor et al., 2000). The profuse glial processes appear interposed between the presynaptic and the postsynaptic membrane, as has been observed at the electron microscopy level, decreasing or suppressing synaptic transmission, a fact that contributes to the loss of input signals and, therefore, to alterations in the discharge pattern (Delgado-Garcia et al., 1988; de la Cruz et al., 2000; Pastor et al., 2000). In line with these arguments, we have observed in axotomized abducens motoneurons a significant increase in GFAP-IR processes and a parallel decrease in synaptophysin-IR synaptic boutons, both around motoneuron somata and in the neuropil. A remarkable finding of the present study was that a single VEGF administration was able to preserve astrocytic GFAP and synaptophysin labeling within normal values, in congruence with the physiological data showing a normal firing pattern in motoneurons, despite their axotomy. In agreement with our data, previous works have also reported the ability of VEGF to decrease the astrocytic reaction that follows different types of injuries in the CNS (Tovar-y-Romo et al., 2007; Zheng et al., 2007; Park et al., 2018; Cabezas et al., 2019).

### Intraventricular administration of VEGF does not induce an angiogenic response in the abducens nucleus

The neuroprotective effects of VEGF have been explained based on two possible mechanisms of action. On the one hand, due to its angiogenic activity, VEGF could provide injured neurons with a better supply of oxygen and nutrients enhancing their chances for recovery. On the other hand, VEGF could act directly as a neurotrophic factor on neurons activating signaling pathways that ultimately restore them to their normal physiological state. To investigate between these two possibilities, we performed a study on the vascular response to VEGF application in the abducens nucleus, at 21 d postlesion and post-VEGF.

Our data showed that, after VEGF administration, there was no change in the vasculature of the abducens nucleus, as compared with controls. We measured, by stereology, vascular density, vessel diameter and the number of vessels per unit area, using GLUT-1-IR profiles as a marker of blood vessels, within the abducens nucleus (identified by ChAT immunolabeling) in control, axotomized, and VEGF-treated animals. We found that neither axotomy nor VEGF treatment induced any change in these vascular parameters in the abducens nucleus, as compared with the control situation. Moreover, we extended our angiogenic study to other brainstem structures and also found absence of vascular changes due to axotomy or VEGF administration. Other authors have also found no signs of vascular response to VEGF treatment. Thus, the exogenous administration of this factor using osmotic pumps or through the internal carotid artery following different types of lesion does not induce the growing of vascular structures (Manoonkitiwongsa et al., 2004; Tovar-y-Romo et al., 2007; Nicoletti et al., 2008). Moreover, it has been demonstrated that the angiogenic activity of VEGF is only promoted by high doses (60 μg/7 d in rats), which, in turn, are not neuroprotective since immature blood vessels formed by angiogenesis are more permeable leading to tissue edema (Manoonkitiwongsa et al., 2004).

In conclusion, and due to the potentiality of VEGF for the treatment of motoneuron disorders, the long-lasting physiological effects of a single dose of VEGF after motoneuron injury found in the present work raise expectations for therapeutic administration, which would avoid the side effects of repetitive or high doses of VEGF.

## Synthesis

Reviewing Editor: Dritan Agalliu, Columbia University Medical Center Decisions are customarily a result of the Reviewing Editor and the peer reviewers coming together and discussing their recommendations until a consensus is reached. When revisions are invited, a fact-based synthesis statement explaining their decision and outlining what is needed to prepare a revision will be listed below. The following reviewer(s) agreed to reveal their identity: Jeffrey Loeb. Note: If this manuscript was transferred from JNeurosci and a decision was made to accept the manuscript without peer review, a brief statement to this effect will instead be what is listed below.

The study by Calvo and colleagues revisit the effects of VEGF administration following axotomy in adult cat motor neurons. The benefits of chronic VEGF administration on axotomized motor neurons has been previously reported by the same group. However, the novelty of this study is that a single dose administered intraventricularly of VEGF has similar rescue benefits to those reported by chronic administration. This is quite an important finding, particularly with respect to potential therapeutic treatments in pathological and/or neurodegenerative situations.

Reviewer # 1

The study has been well controlled and presented in a succinct manner. However, there are several minor points that the authors need to address:

1. In Figure 2, the authors show the firing rate from a control, axotomized motor neuron after 18 days of axotomy and a motor neuron treated with VEGF following 26 and 38 days after axotomy. It will be beneficial if the authors show the firing rate from an aged-matched axotomized motor neuron (either at 26 days or 38 days after axotomy and treated with VEGF). In this manner, direct comparison between axotomy alone and axotomy+VEGF treatment would be preferential.

2. The firing rate analysis shown in Figure 2E and 2F includes two motor neurons with VEGF shown in different shades of green in the graphs. This is too difficult to discern. The authors should change the shades of green into two distinct colors to differentiate between VEGF-treated motor neurons.

3. It will be very helpful to add the individual points (together with bar charts) in Figure 3E and 3F in order to show the variability in the Ks and Rs for the three experimental groups.

4. Could the authors replace the images in Figure 5F and 5G, since in these images are very few motor neurons are shown (in comparison) with 5E. In this way, the synaptophysin profiling will be better compared for the relevant area within the motor neuron nucleus across the three experimental groups.

5. The title of Figure 5C should read: Axotomy+VEGF (instead of VEGF; since this implies that VEGF was administered in control animals).

6. In line 74, the authors are encouraged to state which neurological diseases are referring to.

7.The Discussion can also be improved. As it stands, it summarizes in most parts the results of the study. I would have like to see a more thorough and in depth discussion about the mechanisms involved of the rescue effects of axotomized motor neurons following SINGLE administration of VEGF compared to the chronic treatment. Although the authors have made parallels with other neurotrophic factors, I would have encouraged the authors to even speculate about the long term benefits of a single administration of VEGF on the function of motor neurons.

Reviewer # 2

This is an interesting study that is generally very well done and convincing showing maintained physiological, synaptic staining, and lack of glial activation around abducens nuclei motor neurons when a single intraventricular dose of VEGF is given at the time of VI nerve transection.

Major concerns:

1) A major conclusion, however, does not seem to be well supported that VEGF signaling on motor neurons is responsible for these beneficial effects. Specifically, stating that there is no effect of the vasculature is inconsistent with the photos they presented and needs a more detailed analysis. While changes in vasculature may not be consistent with their hypothesis, this may be a valuable way to promote neuroprotection and needs to be explored in more detail to improve the mechanistic aspect of this study.

2) A second, significant concern is the similarity in results and figures with their paper published in 2018. In fact, some of the confocal images look like different planes of the same neurons. As such, there are few new findings other than the ability of a single dose to have long-lasting effects that were achieved previously with more frequent dosing delivered to the nerve stump.

Minor Concerns:

1) The Glut1 staining is reported to be unchanged after axotomy/rescue with VEGF. I strongly disagree based on the photos provided.

2) The control has many more smaller vessels. After axotomy larger diameter vessels appear and these are also seen in the VEGF. The control has small vessels in close proximity to the motor neurons. this is lost in the axotomy and partially restored with VEGF.

3) VEGF treatment seems to have more small diameter vessels in close approximation to the motor neurons than the untreated/axotomy animals.

4) The quantitation needs to be redone in a blinded way to address these points and develop quantitative metrics for each. this should also be repeated in another region of the brainstem outside the abducens nucleus to see if this effect is a general effect or one that is specific for axotomized motor neurons.

5) Clearly the crude area/area method used was insufficient to see these effects that are clearly visible and of crucial importance for the interpretation of their other interesting results.

6) No mention was made on whether the quantitation of fluorescent images, including the linear density of synaptic labeling and vascular density, was blinded to treatment/controls to rule out subjective bias.

7) One additional mechanism to consider about the long term effects of VEGF vs other neurotrophic factors could be its heparin-binding capabilities that allow it to penetrate better and remain in the ECM, similar to the neuregulin works. Repeating the study with the truncated form of VEGF (120) could validate this hypothesis.
